# Identification of potential biomarkers of cuproptosis in cerebral ischemia

**DOI:** 10.3389/fnut.2024.1410431

**Published:** 2024-09-18

**Authors:** Lihua Qin, Xi Cao, Tengjia Huang, Yixin Liu, Sheng Li

**Affiliations:** ^1^School of Nursing, Hunan University of Chinese Medicine, Changsha, Hunan, China; ^2^Key Laboratory of Hunan Province for Prevention and Treatment of Integrated Traditional Chinese and Western Medicine on Cardiocerebral Diseases, Hunan University of Chinese Medicine, Changsha, Hunan, China

**Keywords:** cerebral ischemia, cuproptosis, biomarkers, gene expression, bioinformatics

## Abstract

**Objective:**

Cerebral ischemia can cause mild damage to local brain nerves due to hypoxia and even lead to irreversible damage due to neuronal cell death. However, the underlying pathogenesis of this phenomenon remains unclear. This study utilized bioinformatics to explore the role of cuproptosis in cerebral ischemic disease and its associated biomarkers.

**Method:**

R software identified the overlap of cerebral ischemia and cuproptosis genes, analyzed Gene Ontology (GO) and Kyoto Encyclopedia of Genes and Genomes (KEGG), and explored hub genes. Expressions and localizations of hub genes in brain tissue, cells, and immune cells were analyzed, along with predictions of protein structures, miRNAs, and transcription factors. A network was constructed depicting hub gene co-expression with miRNAs and interactions with transcription factors. Ferredoxin 1 (*FDX1*) expression was determined using qRT-PCR.

**Results:**

Ten cuproptosis-related genes in cerebral ischemia were identified, with GO analysis revealing involvement in acetyl-CoA synthesis, metabolism, mitochondrial function, and iron–sulfur cluster binding. KEGG highlighted processes like the tricarboxylic acid cycle, pyruvate metabolism, and glycolysis/gluconeogenesis. Using the Human Protein Atlas, eight hub genes associated with cuproptosis were verified in brain tissues, hippocampus, and AF22 cells. Lipoyl(octanoyl) transferase 1 (*LIPT1*), was undetected, while others were found in mitochondria or both nucleus and mitochondria. These genes were differentially expressed in immune cells. *FDX1*, lipoic acid synthetase (*LIAS*), dihydrolipoamide *S*-acetyltransferase (*DLAT*), pyruvate dehydrogenase E1 component subunit alpha 1 (*PDHA1*), *PDHB*, and glutaminase (*GLS*) were predicted to target 111 miRNAs. *PDHA1*, *FDX1*, *LIPT1*, *PDHB*, *LIAS*, *DLAT, GLS*, and dihydrolipoamide dehydrogenase (*DLD*) were predicted to interact with 11, 10, 10, 9, 8, 7, 5, and 4 transcription factors, respectively. Finally, FDX1 expression was significantly upregulated in the hippocampus of ovariectomized rats with ischemia.

**Conclusion:**

This study revealed an association between cerebral ischemic disease and cuproptosis, identifying eight potential target genes. These findings offer new insights into potential biomarkers for the diagnosis, treatment, and prognosis of cerebral ischemia, and provide avenues for the exploration of new medical intervention targets.

## Background

Cerebral ischemia involves complex pathophysiological processes in cells, such as oxidative stress, calcium overload, mitochondrial damage, and excitatory amino acid toxicity. These processes can activate cell death pathways, including apoptosis, programmed necrosis, autophagy, ferroptosis, and pyroptosis (1, 2). Recently, copper-induced cell death, termed cuproptosis, has been established as a novel form of cell death, differing from other programmed cell death mechanisms. Cuproptosis is induced by the binding of copper ions to thioacylated proteins in the tricarboxylic acid (TCA) cycle. This binding leads to abnormal oligomerization of thioacylated proteins, downregulation of Fe-S cluster protein levels, protein toxicity stress, and ultimately cell death (3).

Previous studies have demonstrated that copper ions are involved in the onset and progression of ischemic stroke (IS) (4, 5). Furthermore, plasma copper levels are positively correlated with the risk of initial IS (6, 7), and elevated plasma copper levels are significantly associated with an increased risk of IS (8). These findings underscore the importance of better understanding the relationship between cerebral ischemia and cuproptosis. However, cuproptosis biomarkers in cerebral ischemia have not been fully characterized. Therefore, this study aimed to investigate the hub genes and biomarkers related to cuproptosis in cerebral ischemia using bioinformatics. Furthermore, the research has not only preliminarily validated through Human Protein Atlas (HPA), but also further experimentally validated in model animals to ensure the accuracy and reliability of the research conclusions.

## Methods

### Intersection of cerebral ischemia and cuproptosis datasets

Select “Diseases” through keyword search, enter “cerebral ischemia,” and click search, cerebral ischemia-related genes were downloaded from the Comparative Toxicogenomics Database (CTD).^1^ Ten genes associated with cuproptosis, namely ferredoxin 1 (*FDX1*), lipoyl(octanoyl) transferase 1 (*LIPT1*), lipoic acid synthetase (*LIAS*), dihydrolipoamide dehydrogenase (*DLD*), metal regulatory transcription factor 1 (*MTF1*), dihydrolipoamide S-acetyltransferase (*DLAT*), pyruvate dehydrogenase E1 component subunit alpha 1 (*PDHA1*), *PDHB*, glutaminase (*GLS*), and cyclin-dependent kinase inhibitor 2A (*CDKN2A*), were identified in a previous study by Tsvetkov et al. (3). The genes obtained from both datasets were intersected and visualized using jvenn online tool.^2^

### Gene Ontology (GO)/Kyoto Encyclopedia of Genes and Genomes (KEGG) analyses

The GO/KEGG analyses were performed using clusterProfiler (4.4.4) packages in R (4.2.1) software. The ID conversion of the input molecular lists was converted via the org.Hs.eg.db package in the ID conversion library (9). Enrichment analysis and visualization were performed using the ggplot2 (3.3.6), igraph (1.4.1), and ggraph packages (2.1.0) (10).

### Hub gene analysis

Protein interactions were analyzed using the STRING Database (Search Tool for the Retrieval of Interacting Genes/Proteins; version 11.0, http://string-db.org) (11). The gene cluster was analyzed using the maximum clique centrality and degree methods (MCODE) plug-in of the Cytoscape software (3.7.2). The full names and descriptions of the hub genes were extracted from Gene Cards.^3^

### Expression, localization, and protein structure of hub genes in brain tissues, brain cells, and immune cells

The expression of hub genes was verified using the HPA online database in brain tissues, human neuroepithelial stem cells (AF22 cells), and immune cells.^4^ Subcellular localization of hub genes and prediction of their protein structure are shown. Regarding the confidence of the predicted protein structure, deep blue is used to indicate very high confidence (pLDDT >90), light blue is used to indicate high confidence (90 > pLDDT >70), yellow is used to indicate low confidence (70 > pLDDT >50), and orange is used to indicate very low confidence (pLDDT <50) (see Footnote 4).

### Co-expression network of hub genes and micro RNAs (miRNAs)

The target miRNAs associated with hub genes related to cuproptosis were predicted using the miRTarBas9.0 database,^5^ and two validation methods were selected to verify the predicted target miRNAs. Subsequently, a co-expression network was constructed using Cytoscape software.

### Transcription factor (TF)–gene interactions

The TFs of hub genes related to cuproptosis were searched using the NetworkAnalyst database.^6^ The JASPAR database^7^ was selected as the TF and gene interaction database, and the interaction relationships were visualized using the JASPAR database (see Footnote 7).

### Quantitative real-time polymerase chain reaction (qRT-PCR)

Previous studies on cerebral ischemia often used male rats to minimize estrogen influence. However, the soaring incidence of IS among postmenopausal women (12), with enlarged infarction and aggravated brain damage in ovariectomized rats (13), poses a critical threat. Our study employed female rats with ovariectomy followed by cerebral ischemia induction for animal experimentation.

According to body weight, 8-week-old female SD rats were randomly divided into sham and model groups, each group with three rats. Rats were adapted to an SPF environment at the Experimental Animal Center of Hunan University of Chinese Medicine for 1 week. Referring to previous literature (14), the ovariectomy was performed and the cerebral ischemia model was prepared, the specific steps are as follows: the rats were fasted for 12 h before surgery and anesthetized with 2% pentobarbital sodium via intraperitoneal injection. The model group rats quickly made approximately 3 cm incisions on both sides of their backs under sterile conditions, and after tightening the fallopian tubes, the ovaries were removed and the incisions were sutured. The back skin of rats in the sham group was cut open, bilateral ovaries were separated, and then directly sutured. After surgery, rats were administered with 160,000 units of penicillin via intraperitoneal injection daily for a total of 3 days. Starting from the 5th day post-surgery, vaginal smears were collected from the rats once daily for a total of 5 days. The success of modeling was determined by the absence of an estrous cycle response during this period. Starting from the 5th day after surgery, vaginal secretion smears from rats were taken for smear testing once a day for a total of 5 days, and the successful modeling was considered as no occurrence of estrous cycle reactions. After the successful ovariectomy model in rats, fasting for 12 h was performed. On the 12th day post-surgery, they were anesthetized by intraperitoneal injection of 2% pentobarbital sodium. A midline incision was made on the neck, blunt dissection was performed, and the right common carotid artery was exposed. The internal and external carotid arteries were separated, and the proximal and distal ends of the external carotid artery were ligated. A monofilament nylon suture was inserted into the internal carotid artery to a depth of (18.5 ± 0.5) mm until slight resistance was felt, and then the suture was fixed. The incision was sutured layer by layer. Neurological function was assessed using the Longa score 24 h after cerebral ischemia, with scores of 1 to 3 indicating a successful model. The skin of the rats in the sham surgery group was cut open, and the right common carotid artery was separated, and sutured.

After successful modeling, the rats were anesthetized via intraperitoneal injection of 2% pentobarbital sodium and sacrificed, and their hippocampal tissue was collected. Total RNA was extracted and synthesized into cDNA using the total RNA Extraction Kit (Jiangsu Cowin Biotech Co., Ltd., CW0581S) and qRT-PCR. The expression of *FDX1* was assessed using the 2^-ΔΔCt^ method (forward: AAGAACCGAGATGGTGAAAC, reverse: AGAGCAAGCCAAAGTCCC, 126 bp).

### Statistical analysis

The data calculations and statistical analysis were performed using IBM SPSS Statistics 25.0. Independent sample *t*-test was used for intergroup comparisons (*p <* 0.05).

## Results

**Figure 1:**
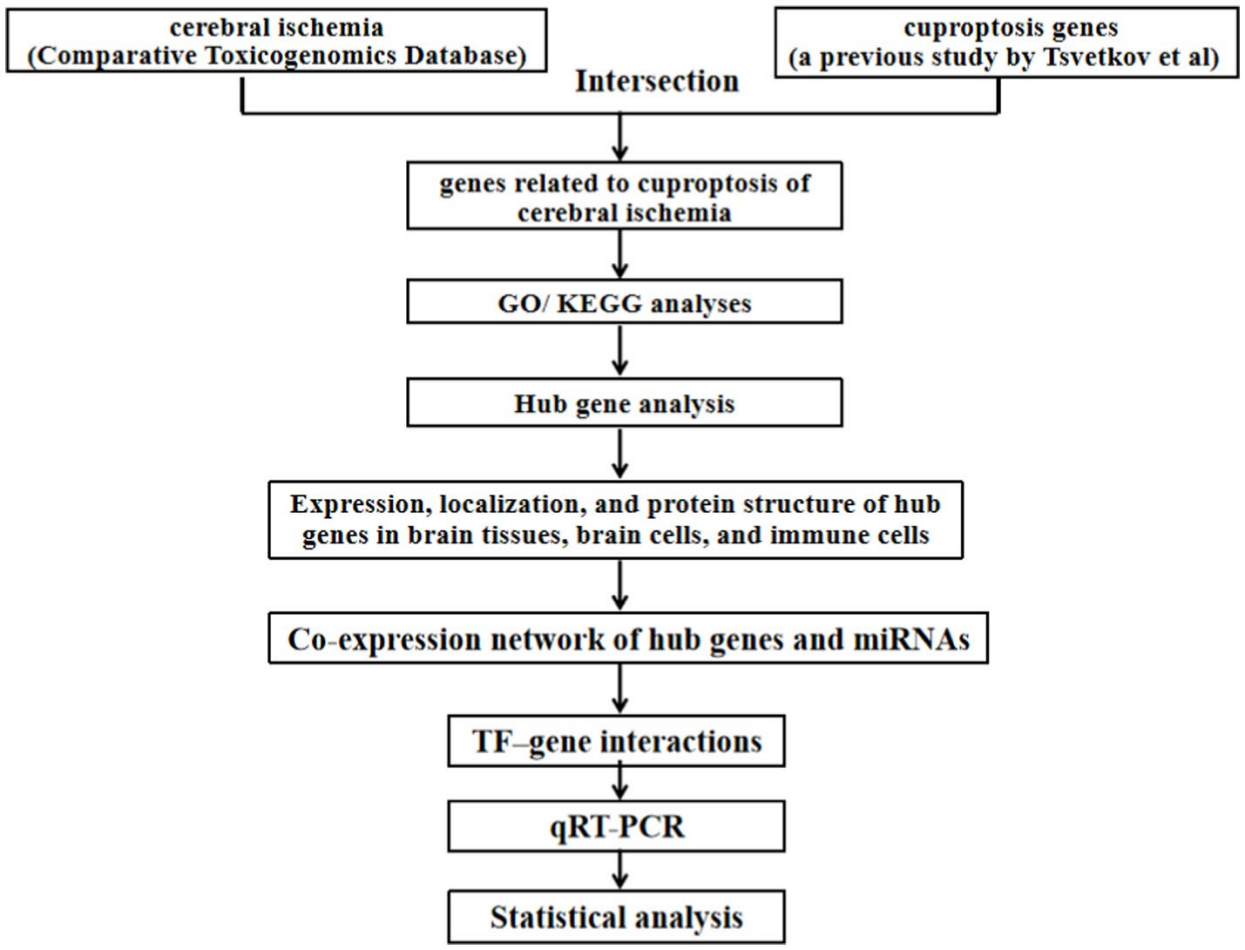
Flowchart of this study.

The flowchart of this study was shown in Figure 1.

### GO and KEGG enrichment analyses for cuproptosis genes in cerebral ischemia

**Figure 2:**
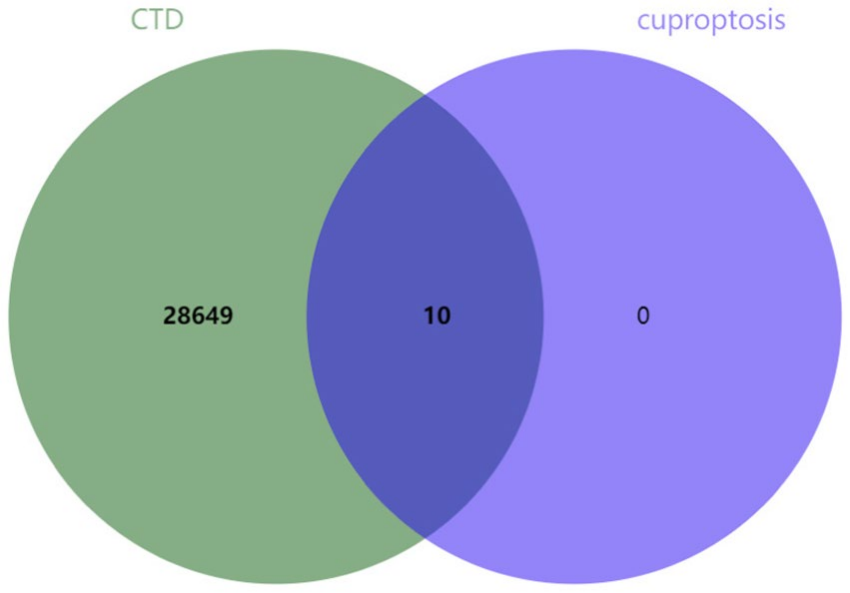
Common genes in cerebral ischemia and cuproptosis.

Ten genes (*FDX1*, *LIPT1*, *LIAS*, *DLD*, *MTF1*, *DLAT*, *PDHA1*, *PDHB*, *GLS*, and *CDKN2A*) associated with cuproptosis were intersected with genes related to brain ischemia, revealing that all 10 genes were brain ischemia-related genes (refer to Figure 2).

**Figure 3:**
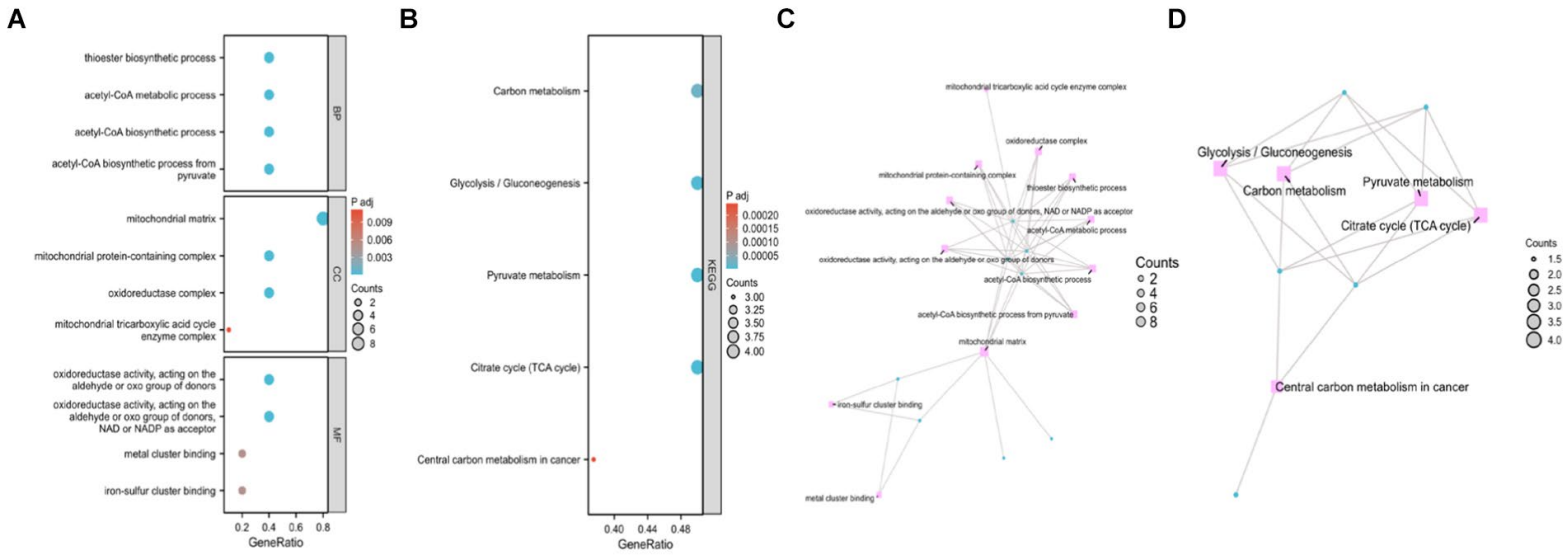
Gene Ontology (GO) and Kyoto Encyclopedia of Genes and Genomes (KEGG) enrichment analyses of genes related to cuproptosis in cerebral ischemia. **(A)** Bubble Chart of top four enriched biological process (BP) terms, cellular components (CCs), and molecular function (MF) terms for genes related to cuproptosis on cerebral ischemia. **(B)** Bubble Chart of top five KEGG pathways for genes related to cuproptosis in cerebral ischemia. **(C)** Network diagram of top four enriched BP, (CC), and MF terms for genes related to cuproptosis in cerebral ischemia. **(D)** Network diagram of five KEGG pathways of genes related to cuproptosis in cerebral ischemia.

The significance cut-off value for the GO/KEGG analyses was set at a corrected *p*-value (*p*.adj) of <0.05. The results of the identified biological processes (BPs), cellular components (CCs), and molecular functions (MFs) were 81, 6, and 15, respectively. KEGG analysis revealed enrichment in 8 pathways. The top 5 GO and KEGG pathways are presented in Tables 1, 2, as well as Figure 3.

**Table 1:** Biological process, cellular composition, and molecular function analysis results for genes related to cuproptosis in cerebral ischemia.

Ontology	ID	Description	GeneRatio	BgRatio	*p*-value	*p*.adjust	geneID
BP	GO:0006086	Acetyl-CoA biosynthetic process from pyruvate	4/10	11/18800	1.33e-11	2.53e-09	DLAT/PDHB/DLD/PDHA1
BP	GO:0006085	Acetyl-CoA biosynthetic process	4/10	18/18800	1.23e-10	1.17e-08	DLAT/PDHB/DLD/PDHA1
BP	GO:0006084	Acetyl-CoA metabolic process	4/10	33/18800	1.64e-09	1.04e-07	DLAT/PDHB/DLD/PDHA1
BP	GO:0035384	Thioester biosynthetic process	4/10	45/18800	5.95e-09	2.26e-07	DLAT/PDHB/DLD/PDHA1
BP	GO:0071616	Acyl-CoA biosynthetic process	4/10	45/18800	5.95e-09	2.26e-07	DLAT/PDHB/DLD/PDHA1
CC	GO:0005759	Mitochondrial matrix	8/10	473/19594	4.69e-12	3.75e-11	DLAT/PDHB/GLS/LIPT1/FDX1/DLD/LIAS/PDHA1
CC	GO:1990204	Oxidoreductase complex	4/10	120/19594	2.73e-07	1.09e-06	DLAT/PDHB/DLD/PDHA1
CC	GO:0098798	Mitochondrial protein-containing complex	4/10	281/19594	8.12e-06	2.17e-05	DLAT/PDHB/DLD/PDHA1
CC	GO:0030062	Mitochondrial tricarboxylic acid cycle enzyme complex	1/10	11/19594	0.0056	0.0112	DLD
CC	GO:0045239	Tricarboxylic acid cycle enzyme complex	1/10	16/19594	0.0081	0.0130	DLD
MF	GO:0016620	Oxidoreductase activity, acting on the aldehyde or oxo group of donors, NAD or NADP as acceptor	4/10	38/18410	3.21e-09	1.06e-07	DLAT/PDHB/DLD/PDHA1
MF	GO:0016903	Oxidoreductase activity, acting on the aldehyde or oxo group of donors	4/10	46/18410	7.08e-09	1.17e-07	DLAT/PDHB/DLD/PDHA1
MF	GO:0051536	Iron–sulfur cluster binding	2/10	67/18410	0.0006	0.0048	FDX1/LIAS
MF	GO:0051540	Metal cluster binding	2/10	67/18410	0.0006	0.0048	FDX1/LIAS
MF	GO:0016783	Sulfurtransferase activity	1/10	10/18410	0.0054	0.0269	LIAS

**Table 2:** Kyoto Encyclopedia of Genes and Genomes analysis results for genes related to cuproptosis in cerebral ischemia.

Ontology	ID	Description	GeneRatio	BgRatio	*p*-value	*p*.adjust	geneID
KEGG	hsa00020	Citrate cycle (TCA cycle)	4/8	30/8164	1.03e-08	3.8e-07	DLAT/DLD/DLST/PDHA1/PDHB
KEGG	hsa00620	Pyruvate metabolism	4/8	47/8164	6.64e-08	1.23e-06	DLAT/DLD/DLST/GCSH/PDHA1/PDHB
KEGG	hsa00010	Glycolysis/Gluconeogenesis	4/8	67/8164	2.83e-07	3.49e-06	DLAT/DLD/PDHA1/PDHB
KEGG	hsa01200	Carbon metabolism	4/8	115/8164	2.5e-06	2.32e-05	DLAT/DLD/PDHA1/PDHB
KEGG	hsa05230	Central carbon metabolism in cancer	3/8	70/8164	3.28e-05	0.0002	GLS/PDHA1/PDHB

Enriched BPs included acetyl-CoA biosynthetic process from pyruvate, acetyl-CoA metabolic process, acetyl-CoA metabolic process, acetyl-CoA biosynthetic process, acetyl-CoA biosynthetic process, and thioester biosynthetic process. CCs encompassed various complexes, such as mitochondrial matrix, oxidoreductase complex, mitochondrial protein-containing complex, mitochondrial tricarboxylic acid cycle enzyme complex, and tricarboxylic acid cycle enzyme complex. MFs included activities such as oxidoreductase activity, acting on the aldehyde or oxo group of donors, NAD or NADP as acceptor, oxidoreductase activity, acting on the aldehyde or oxo group of donors, iron–sulfur cluster binding, metal cluster binding, and sulfurtransferase activity. Moreover, KEGG analysis revealed involvement in processes such as pyruvate metabolism, citrate cycle (TCA cycle), carbon metabolism, glycolysis/gluconeogenesis, and central carbon metabolism in cancer.

### Cuproptosis-related hub gene identification and their expression in the brain

**Figure 4:**
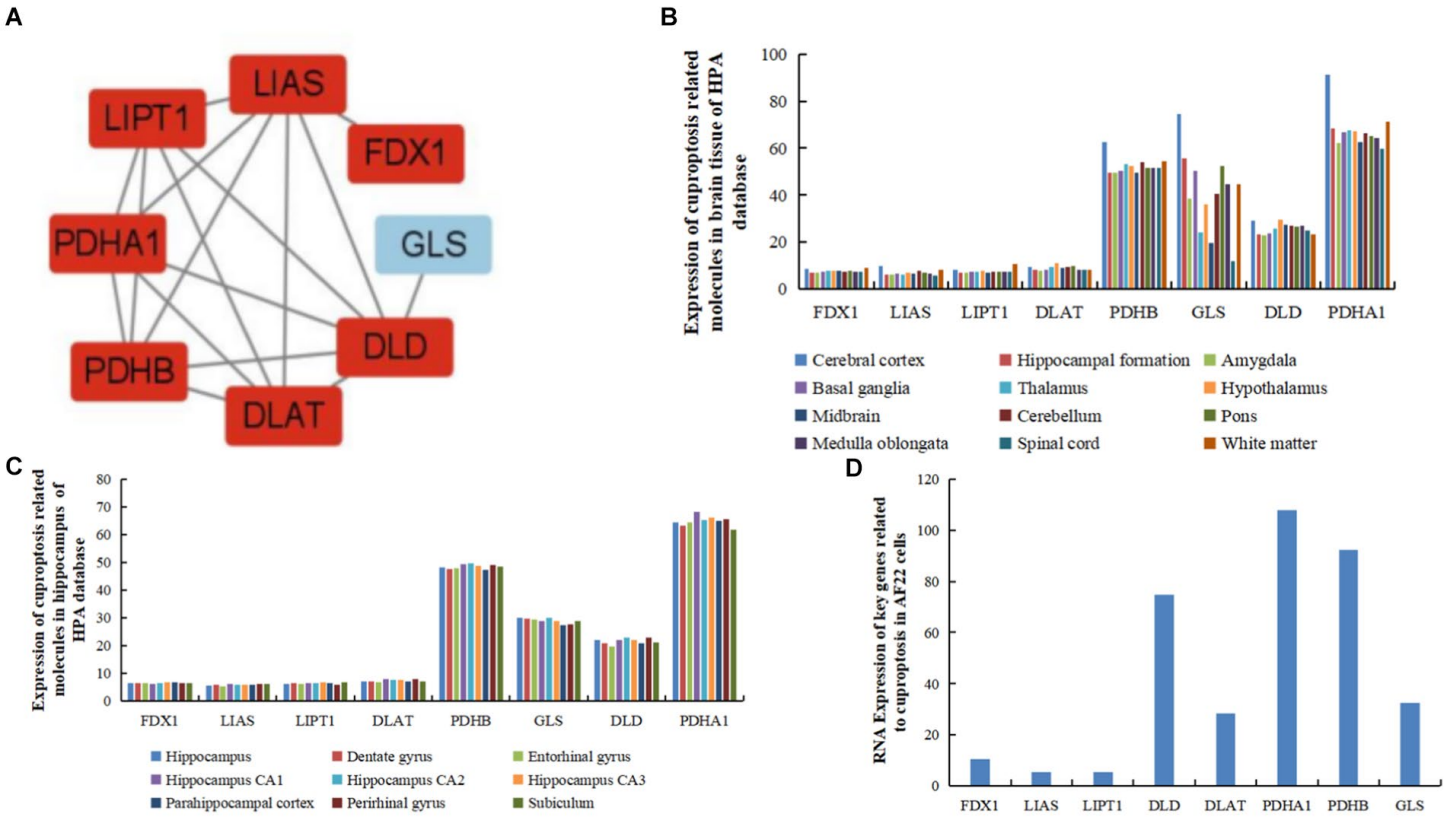
Expression of hub genes related to cuproptosis and cerebral ischemia in brain tissues, and human and AF22 cells. **(A)** Hub genes of genes related to cuproptosis in cerebral ischemia identified using MCODE. **(B)** Expression of hub genes related to cuproptosis in brain tissues in the HPA database. **(C)** Expression of hub genes related to cuproptosis in the hippocampus in the HPA database. **(D)** RNA Expression of hub genes related to cuproptosis in human neuroepithelial stem cells (AF22 cells).

Eight hub genes (*FDX1*, *LIPT1*, *LIAS*, *DLD*, *PDHA1*, *DLAT*, *PDHB*, and *GLS*) were identified as potential core targets of cerebral ischemia (Figure 4A and Table 3). Using the HPA database (see footnote 4), the expression of the hub genes was analyzed in brain tissues, revealing that these genes associated with cuproptosis were expressed in various regions of the human brain, such as the cortex, hippocampus, amygdala, basal ganglia, thalamus, hypothalamus, midbrain, white matter of the spinal cord, and other areas (Figure 4B). Furthermore, these genes were expressed in specific regions within the internal structure of the hippocampus (Figure 4C).

**Table 3:** Details of the hub genes related to cuproptosis in cerebral ischemia.

Gene symbol	Full name	Description
FDX1	Ferredoxin 1	This gene encodes a small iron–sulfur protein that transfers electrons from NADPH through ferredoxin reductase to mitochondrial cytochrome P450, involved in steroid, vitamin D, and bile acid metabolism.
LIAS	Lipoic acid synthetase	The protein encoded by this gene belongs to the biotin and lipoic acid synthetases family. Localized in the mitochondrion, this iron–sulfur enzyme catalyzes the final step in the *de novo* pathway for the biosynthesis of lipoic acid, a potent antioxidant.
LIPT1	Lipoyltransferase 1	The process of transferring lipoic acid to proteins is a two-step process. The first step is the activation of lipoic acid by lipoate-activating enzyme to form lipoyl-AMP. For the second step, the protein encoded by this gene transfers the lipoyl moiety to apoproteins. Alternative splicing results in multiple transcript variants.
DLD	Dihydrolipoamide dehydrogenase	This gene encodes a member of the class-I pyridine nucleotide-disulfide oxidoreductase family. The encoded protein has been identified as a moonlighting protein based on its ability to perform mechanistically distinct functions.
DLAT	Dihydrolipoamide *S*-acetyltransferase	This gene encodes component E2 of the multi-enzyme pyruvate dehydrogenase complex (PDC). PDC resides in the inner mitochondrial membrane and catalyzes the conversion of pyruvate to acetyl coenzyme A.
PDHA1	Pyruvate dehydrogenase E1 subunit alpha 1	The pyruvate dehydrogenase (PDH) complex is a nuclear-encoded mitochondrial multienzyme complex that catalyzes the overall conversion of pyruvate to acetyl-CoA and CO(2), and provides the primary link between glycolysis and the tricarboxylic acid (TCA) cycle.
PDHB	Pyruvate dehydrogenase E1 subunit beta	The pyruvate dehydrogenase (PDH) complex is a nuclear-encoded mitochondrial multienzyme complex that catalyzes the overall conversion of pyruvate to acetyl-CoA and carbon dioxide, and provides the primary link between glycolysis and the tricarboxylic acid (TCA) cycle.
GLS	Glutaminase	This gene encodes the K-type mitochondrial glutaminase. The encoded protein is an phosphate-activated amidohydrolase that catalyzes the hydrolysis of glutamine to glutamate and ammonia.

In human neuroepithelial stem cells (AF22 cells) from the HPA database, *PDHA1* exhibited the highest expression, followed by *PDHB*, *DLD*, *GLS*, *DLAT*, and *FDX1*. In contrast, *LIAS* and *LIPT1* exhibited the lowest expression levels (Figure 4D).

### Subcellular localization and structural prediction of cuproptosis-related hub gene

**Figure 5:**
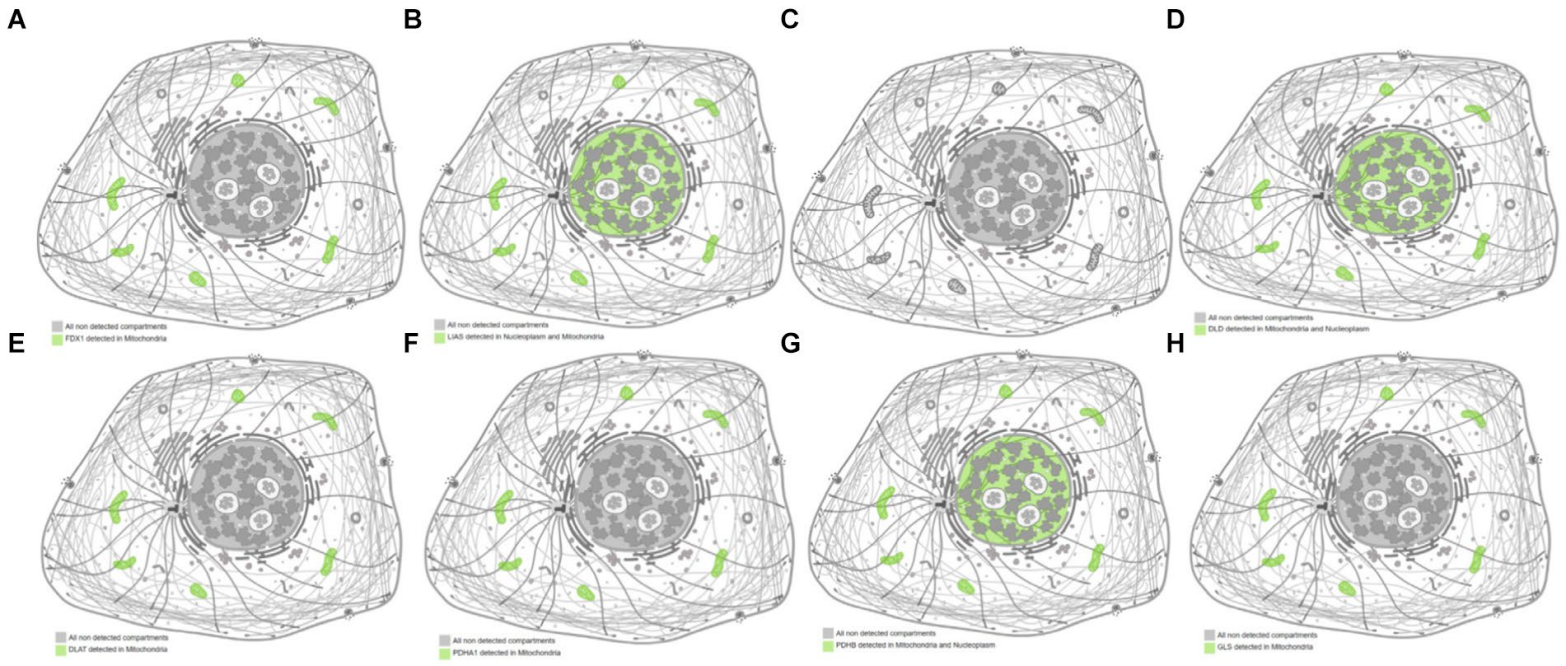
Subcellular localization of hub genes related to cuproptosis and cerebral ischemia. **(A)** FDX1; **(B)** LIAS; **(C)** LIPT1; **(D)** DLD; **(E)** DLAT; **(F)** PDHA1; **(G)** PDHB; **(H)** GLS.

**Figure 6:**
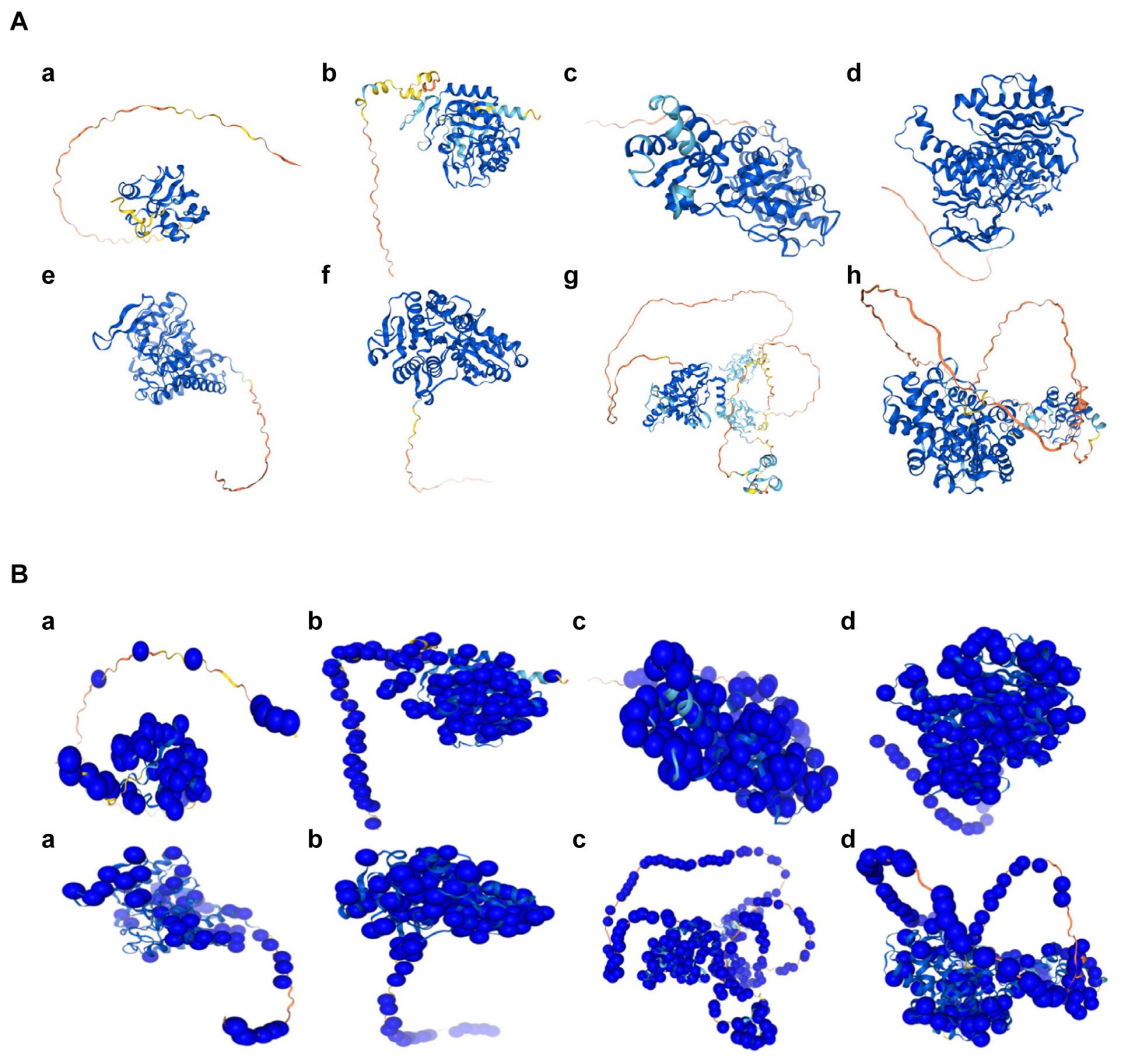
The predicted structures of hub genes related to cuproptosis of cerebral ischemia. **(A)** The predicted structure diagrams, colored based on the confidence score per residue (pLDDT); dark blue represents the highest confidence level, and orange represents the lowest confidence level. a, FDX1; b, LIAS; c, LIPT1; d, DLD; e, PDHA1; f, PDHB; g, *DLAT*; h, GLS. **(B)** The predicted structures of the variable populations. a, FDX1; b, LIAS; c, LIPT1; d, DLD; e, PDHA1; f, PDHB; g, DLAT; h, GLS.

The subcellular localization results indicated that *FDX1*, *DLAT*, *PDHA1*, and *GLS* were detected only in the mitochondria, whereas *LIAS*, *DLD*, and *PDHB* were detected in both the nucleus and mitochondria (Figure 5). The predicted structure diagrams of the hub genes and predicted structures of variant populations are shown in Figures 6A,B, respectively.

### Expression of cuproptosis-related hub genes in immune cells

**Figure 7:**
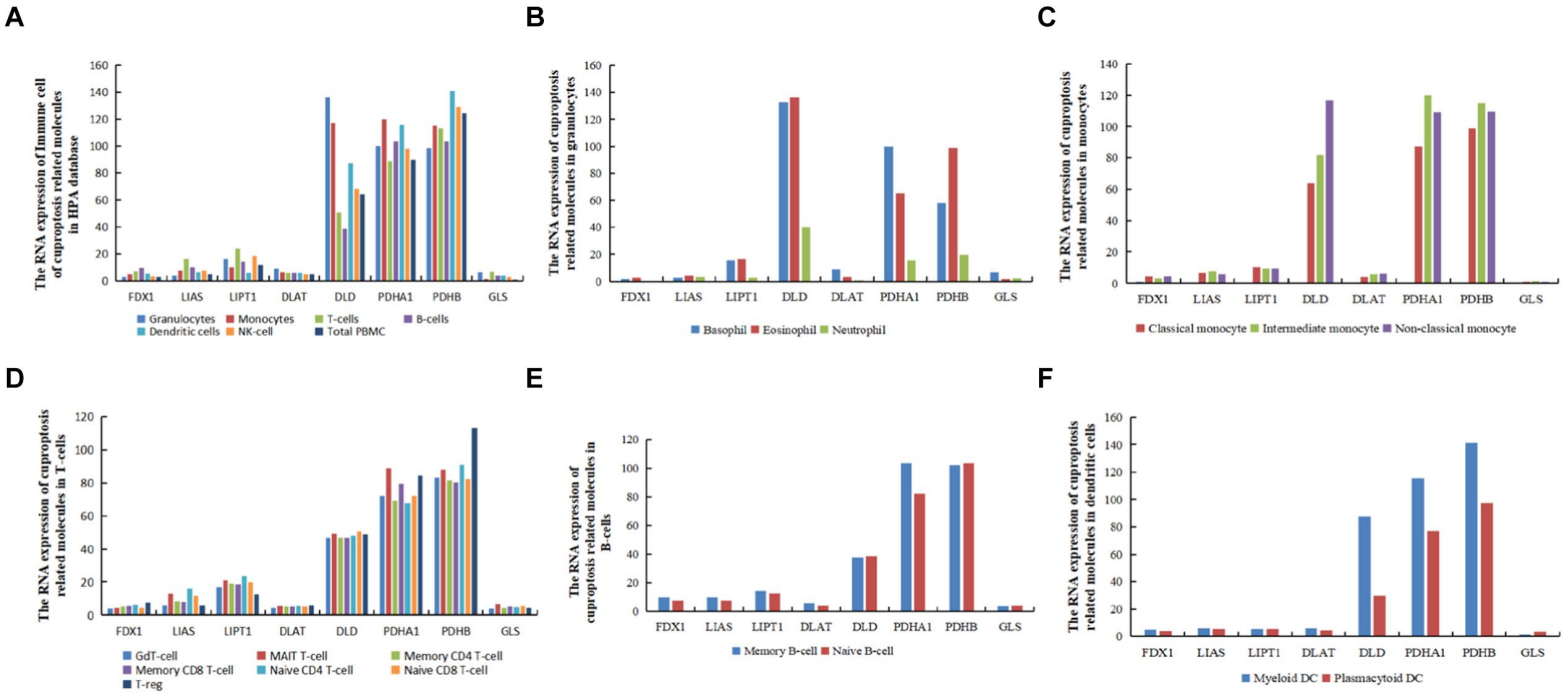
The RNA levels of hub genes related to cuproptosis and cerebral ischemia in immune cells in the HPA database (nTPM, transcripts per million). **(A)** The RNA expression of immune cell of cuproptosis related molecules in HPA database. **(B)** The RNA expression of cuproptosis related molecules in granulocytes. **(C)** The RNA expression of cuproptosis related molecules in monocytes. **(D)** The RNA expression of cuproptosis related molecules in T-cells. **(E)** The RNA expression of cuproptosis related molecules in dendritic B-cells. **(F)** The RNA expression of cuproptosis related molecules in dendritic cells.

The RNA expression data from the immune cells of the HPA database^9^ revealed that *FDX1* exhibited the highest expression in B cells. In contrast, *LIAS*, *LIPT1*, and *GLS* exhibited the highest expression in T cells, *DLAT* and *DLD* in granulocytes, *PDHA1* in monocytes, and *PDHB* in dendritic cells. *FDX1* and *GLS* exhibited the lowest expression levels in peripheral blood mononuclear cells (PBMCs), *LIAS* and *PDHB* in granulocytes, *LIPT1* in dendritic cells, *PDHA1* in T cells, *DLD* in B cells, and *DLAT* in natural killer (NK) cells. Moreover, *FDX*1 and *DLAT* exhibited the lowest expression levels in granulocytes, and *GLS* in monocytes, B cells, NK cells, total PBMC, and dendritic cells (Figure 7A).

In granulocytes, *FDX1*, *LIAS*, *LIPT1*, *DLD*, and *PDHB* exhibited the highest expression in eosinophils, whereas *PDHA1*, *DLAT*, and *GLS* genes were most highly expressed in basophils (Figure 7B). In monocytes, *FDX1*, *DLAT*, and *DLD* were most highly expressed in non-classical monocytes, *LIPT1* was most highly expressed in classical monocytes, and *LIAS,GLS*, *PDHA1*, *LIAS*, and *PDHB* were most highly expressed in intermediate monocytes (Figure 7C). In T cells, *FDX1*, *DLAT*, and *PDHB* were most highly expressed in T regulatory cells (T-regs), *LIAS* and *LIPT1* in naive CD4 T cells, *DLD* in memory CD8 T cells, and *GLS* and *PDHA1* in mucosal-associated invariant (MAIT) T cells (Figure 7D). In B cells, except for *DLD* and *GLS*, which exhibited the highest expression in naive B cells, the remaining six genes were most highly expressed in memory B cells (Figure 7E). In dendritic cells (DCs), except for *GLS*, which was most highly expressed in plasmacytoid DCs, and *LIPT1*, which was equally expressed in myeloid DCs and plasmacytoid DCs, all other genes were most highly expressed in myeloid DCs (Figure 7F).

### Co-expression network of hub genes and miRNAs

**Figure 8:**
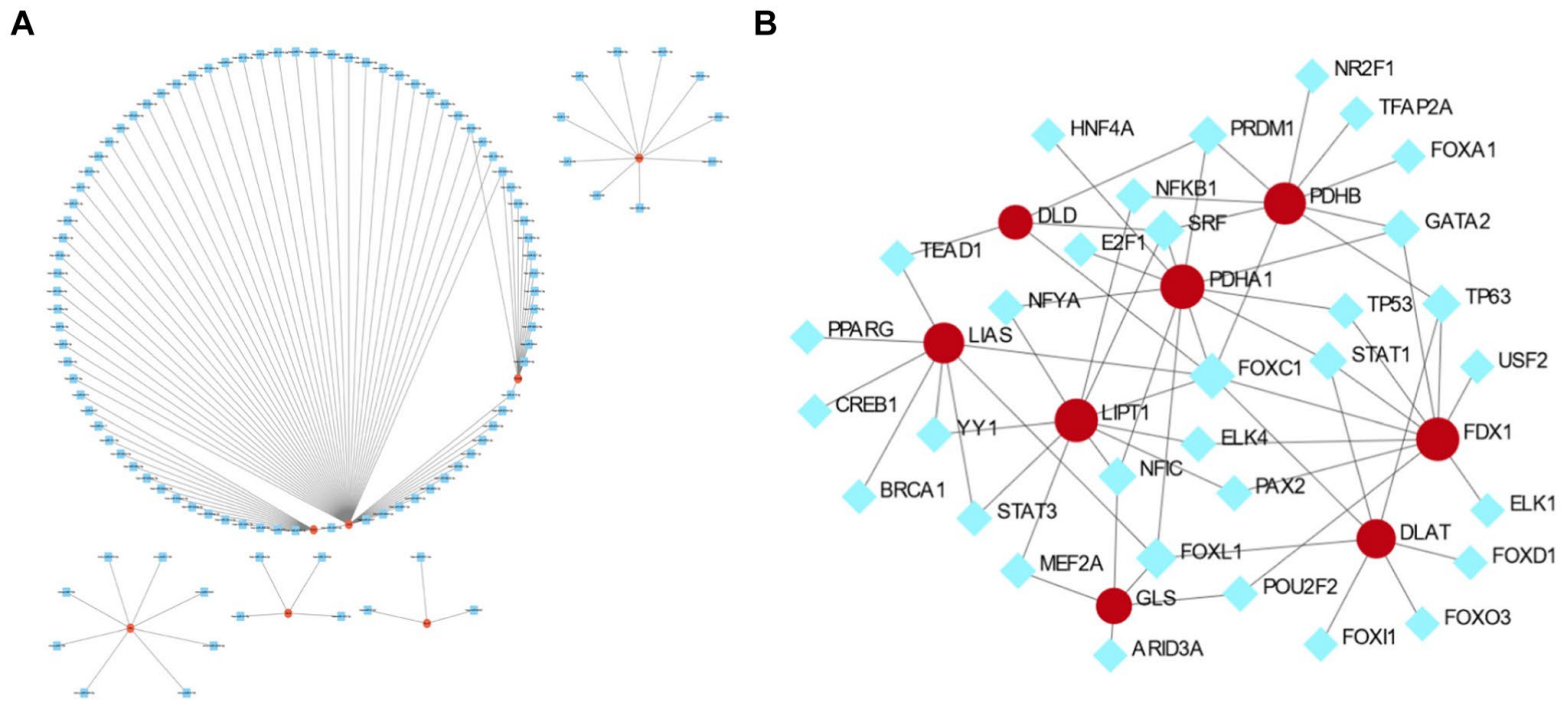
The co-expression network of mRNA–microRNA (miRNA) and transcription factor (TF)–mRNA interactions of hub genes related to cuproptosis in cerebral ischemia. **(A)** The co-expression network of mRNA–miRNA of hub genes related to cuproptosis in cerebral ischemia. **(B)** The TF–mRNA interaction of hub genes related to cuproptosis in cerebral ischemia.

The miRNA targets of the eight cuproptosis-related hub genes were predicted using the miRTarBas9.0 database. Among these genes, six, including *FDX1*, *LIAS*, *DLAT*, *PDHA1*, *PDHB*, and *GLS*, were predicted to target 111 miRNAs. A co-expression network was then constructed using Cytoscape software, as shown in Figure 8A.

### TF–gene interactions

Analysis of TF and gene interactions using the NetworkAnalyst database revealed that *PDHA1*, *FDX1*, *LIPT1*, *PDHB*, *LIAS*, *DLAT*, *GLS*, and *DLD* interact with 11, 10, 10, 9, 8, 7, 5, and 4 TFs, respectively. These interactions are visualized in Figure 8B.

### Expression level of *FDX1*

To further validate the aforementioned results, the expression of *FDX1* was determined through qRT-PCR. Compared to that in the sham group (1.68 ± 0.53), *FDX* expression in the hippocampus of ovariectomized rats with ischemia (5.05 ± 0.03) was significantly upregulated (*t* = −15.58, *p* = 0.00), with 95% confidence interval for the difference (−3.84, −2.88).

## Discussion

Copper is essential for metal signaling regulation, metal allosteric regulation, mitochondrial respiration, antioxidant defense, and neurotransmitter function, also influencing cell fate through metabolic reprogramming (15, 16). Recent studies have revealed that abnormal accumulation of copper ions in human cells induces a distinct form of cell death, distinct from known regulated cell death mechanisms. Copper ions could still trigger cell death even when known cell death modes such as apoptosis, pyroptosis, ferroptosis, and necrotic apoptosis are blocked, relying on mitochondrial respiration (3).

This study identified *FDX1*, *LIAS*, *LIPT1*, *DLD*, *DLAT*, *PDHA1*, *PDHB*, and *GLS* as eight hub genes associated with cuproptosis in cerebral ischemia. Expression analysis in the HPA database revealed that these hub genes were expressed in the hippocampus, amygdala, basal ganglia, thalamus, hypothalamus, midbrain, spinal cord white matter, as well as in different regions within the internal structure of the hippocampus and immune cells.

*FDX1* encodes a small iron–sulfur protein involved in mitochondrial cytochrome reduction, Fe-S cluster biosynthesis, and synthesis of various steroid hormones (17, 18). It serves as a key regulator of cuproptosis (3), primarily localized within the cytosol, endoplasmic reticulum, and nucleus, but its highest activity is observed in the mitochondria (19). The results of this study indicated that its subcellular localization was exclusively in the mitochondria, further elucidating the involvement of specific mitochondrial enzymes in the process of cuproptosis and their relationship with mitochondrial respiration. Moreover, the GO analysis revealed the mitochondrial matrix as the CC, aligning with the subcellular localization predictions and existing literature (19). Previous research demonstrated that *FDX1* knockdown resulted in metabolic alterations, particularly in glucose metabolism, fatty acid oxidation, and amino acid metabolism (7), consistent with the Reactome analysis prediction of the signaling pathway involving the human *FDX1* gene (20). Given that prior literature has conclusively demonstrated and reported FDX1 as a crucial gene in the process of ferroptosis (3), We had prioritized validating the expression of FDX1 through animal experiments in the present study, our research findings indicated an elevated expression level of FDX in the hippocampus of castrated ovariectomized rats with ischemia, which aligns with the previously reported increase of FDX expression in the hippocampus of rats with ischemic injury (21). Overexpression of *FDX1 in vitro* partially reversed the protective effect of dexamethasone (DEX) on rat cerebral infarction, including the DEX-induced significant alleviation of rat cerebral infarction, reduced copper levels, mitochondrial function maintenance, increased GSH levels, and decreased levels of key proteins associated with copper toxicity (21).

The LIAS is primarily expressed in the mitochondria (20), and the present study revealed its localization both in the mitochondria and nucleus. This finding aligns with the CC localization results of the mitochondrial matrix, as shown in the GO analysis. Research indicates that *LIAS* mutations could lead to mitochondrial energy metabolism defects (22). Moreover, LIAS is involved in the synthesis of mitochondria-related metabolic enzymes, the biosynthesis of endogenous fatty acids, energy metabolism, and antioxidant reactions (23, 24). Notably, in the present study, the GO-BP analysis revealed that LIAS participates in complex biosynthetic processes. GO-MF analysis demonstrated the ability of LIAS to bind to iron, sulfur, and metal clusters. FDX1 is a critical upstream regulatory factor for protein thioacylation, which plays a vital role in cells relying on mitochondrial metabolism. FDX1 also acts as a key regulator of steatosis by directly binding to LIAS, thereby exerting a lethal metabolic effect on cancer cells (25). The GO-MF analysis in this study indicated that LIAS can bind to iron–sulfur clusters and metal clusters. Moreover, FDX1 serves as a key regulatory factor for protein lipoylation through direct binding to LIAS and plays a role in metabolic conditioning-induced cell death in cancer cells (25).

LIPT1, an enzyme specific to lipid esters, plays a significant role in copper homeostasis. It is an essential enzyme for activating mitochondrial 2-ketoacid dehydrogenases and is involved in maintaining the oxidative and reductive metabolism of glutamine (26). LIPT1 participates in the biosynthesis and function of lipoic acid and fatty acylation (27, 28). These previous findings align with the results of CC localization in the GO analysis of the present study, indicating mitochondrial matrix as the subcellular localization of LIPT1.

The DLD is an important component of various mitochondrial multienzyme complexes, participating in the composition of complexes such as α-ketoglutarate dehydrogenase, α-ketohexanoate dehydrogenase, and glycine decarboxylase (29). It is also involved in the decarboxylation of pyruvate, converting the product into acetyl-CoA in the TCA cycle (30). The results of this study indicated that DLD was localized in both mitochondria and nuclei. GO-CC analysis results suggested associations with mitochondrial matrix, redox enzyme complex mitochondria, and mitochondrial tricarboxylate cyclase complex. Moreover, KEGG analysis indicated its involvement in pathways such as the TCA cycle and pyruvate metabolism. Research has shown that *DLD* downregulation could affect mitochondrial metabolism, leading to reduced levels of downstream metabolites in the TCA cycle and inducing melanoma cell death (31). Moreover, DLD is known to promote cell death, such as apoptosis (32), copper poisoning-induced cell death (3), and cuproptosis. *DLD* serves as a key gene in cuproptosis and is a positive regulator, enhancing copper-dependent cell death (30). The present study also identified DLD as one of the hub genes responsible for cuproptosis following cerebral ischemia.

DLAT, a mitochondrial protein involved in glucose metabolism, is located in the inner mitochondrial membrane and plays a role in the conversion of pyruvate to acetyl acetyl-CoA (33). Furthermore, *DLAT* overexpression inhibits the production of acetyl-CoA (34). These findings are consistent with the GO/KEGG analyses results of this study. Notably, DLAT is associated with cuproptosis, as copper promotes the oligomerization of DLAT, thereby increasing the insoluble DLAT, leading to protein toxicity stress and cell death (3). This oligomerization is due to the integration of copper with lipoylated proteins in the TCA cycle (3). Cu-induced neuronal degeneration and oxidative damage have been shown to promote the expression of FDX1, DLAT, and HSP70 while reducing that of Fe-S cluster proteins (35). Noteworthily, proteins such as ATP6V1A, DLAT, and HSP70 are expressed in the hippocampus of patients with temporal lobe epilepsy (36). In contrast, patients with acute myocardial infarction exhibit decreased levels of LIAS, PDHB, LIPT1, DLAT, and GLS, along with increased MTF1 levels. A previous Kaplan–Meier analysis indicated the prognostic value of DLAT in ischemic events (37). The present study also identified DLAT as one of the hub genes responsible for cuproptosis after cerebral ischemia.

PDHA1 serves as a crucial component of the pyruvate dehydrogenase (PDH) complex, an enzyme complex that regulates the TCA cycle (38). PDHA1 plays a crucial role in glucose metabolism, oxidative phosphorylation, and the TCA cycle in the mitochondria (39). The results of this study indicated the involvement of PDHA1 in the biosynthesis of acetyl-CoA from pyruvate, the biosynthesis process of acetyl-CoA, and acetyl-CoA metabolism processes. KEGG analysis results indicated that these processes were related to pathways such as the TCA cycle, pyruvate metabolism, and glycolysis/gluconeogenesis. These results are consistent with previous reports (39). In mice, *PDHA1* knockout resulted in ultrastructural disruptions of hippocampal neurons and lactate accumulation (40), which subsequently caused impaired neuronal function (41), ultimately resulting in hippocampal dysfunction. Moreover, prostate cancer cells and human esophageal squamous cancer cells lacking PDHA1 exhibit impaired normal mitochondrial oxidative phosphorylation and a reliance on glycolysis (42, 43). These previous data are consistent with the results of the present study in terms of the subcellular localization to the mitochondria, and the cellular component annotations in GO/KEGG analyses corroborate the preservation of the CC.

The PDHB catalyzes the conversion of pyruvate to acetyl-CoA, bridging the TCA cycle and glycolytic pathway (44). Its expression is predominantly distributed within the mitochondria (45). Consistently, in the present study, the GO-BP results indicated the involvement of PDHB d in the biosynthesis process of acetyl-CoA from pyruvate, the biosynthesis process of acetyl-CoA, and the metabolism of acetyl-CoA. Moreover, KEGG analysis revealed that PDHB participates in BPs related to the TCA cycle, pyruvate metabolism, and glycolysis pathways. In a previous study, *PDHB* knockdown in primary human muscle inhibited pyruvate metabolism and upregulated Ariadne RBR E3 ubiquitin protein ligase 2 (Arih2) in the cellular catabolic pathway (46). Moreover, *dPDHB* knockout shortened the lifespan of adult flies, leading to rough eye phenotypes and abnormal photoreceptor axon targeting (47). Mutations in *PDHA1* and *PDHB* are associated with coenzyme Q10 levels and mitochondrial homeostasis imbalance, severely affecting the brain (48). Currently, research is primarily focusing on the role of PDHB in cancer, particularly in the cuproptosis pathway associated with non-alcoholic fatty liver disease (NAFLD), where both DLD and PDHB are potential candidate genes for NAFLD diagnosis and treatment options (49). Additionally, five genes related to cuproptosis (*FDX1, LIPT1, PDHA1, PDHB,* and *CDKN2A*) are considered candidate biomarkers or therapeutic targets for osteoarthritis synovitis (50). The present study also reported the involvement of PDHB in the cuproptosis pathway in cerebral ischemia.

The GLS catalyzes the hydrolysis of glutamine to produce glutamic acid (50). It exists in mammals in two isoforms: GLS1 and GLS2. GLS1 is primarily expressed in organs such as the brain, heart, pancreas, and kidneys, while GLS2 is mainly expressed in the liver (51). The subcellular localization of GLS1 is in the mitochondria, while GLS2 is located in the nucleus (51). However, the results of this study indicated that GLS was only localized to the mitochondria. Dysregulated expression or dysfunction of GLS results in the overproduction of glutamate, alterations in the expression of inflammatory factors, and the disruption of metabolic homeostasis, resulting in the activation of microglia (51). Upregulation of *GLS1* expression leads to excessive production of glutamate in microglial cells, increasing extracellular glutamate levels, which in turn cause excitotoxicity and neuronal degeneration (51). Notably, cuproptosis is associated with the TCA cycle and the aggregation of lipoylated proteins in the TCA cycle (3, 51). In rats with cerebral artery occlusion, the expression of GLS1 was substantially upregulated, and the GLS inhibitor CB-839 markedly reduced the expression of pro-inflammatory factors, thereby alleviating neuroinflammation and brain damage (52). The present study also identified GLS as a hub gene associated with copper-induced cell death during cerebral ischemia.

This study presents novel insights into hub genes involved in cuproptosis during cerebral ischemia, validating them through multiple approaches including brain tissue, immune cells, localization, structure, and prediction of miRNAs and TFs. However, some limitations should be noted; for example, currently, only one of the hub genes, *FDX1*, has been validated in animal experiments and the hippocamp of rats, while other hub genes and the expression of brain regions need to be validated in experiments. In addition, there is a lack of clinical research on the hub genes associated with cuproptosis. In the future, we will strive to incorporate a wider range of brain regions and multiple gene targets into our research endeavors, further deepening our understanding of the intricate mechanisms underlying ischemic injury. However, in subsequent studies, we are predicting and understanding the binding interactions between small molecules (ligands) and target proteins related to copper death in cerebral ischemia; Using computer simulation technology, small molecule drug molecules are docked to the surface of proteins to search for possible binding sites for drug molecule docking. This part of the research work is currently underway, and once progress is made in the next stage, we will share our findings with everyone.

## Conclusion

In summary, this study utilized genes related to cerebral ischemic and cuproptosis to identify and validate genes implicated in both cerebral ischemic and cuproptosis. Eight hub genes with the potential to serve as novel markers were identified, holding promise as specific biomarkers for the diagnosis, treatment, and prognosis of cerebral ischemia in clinical applications.

## Data Availability

The datasets presented in this study can be found in online repositories. The names of the repository/repositories and accession number(s) can be found in the article/supplementary material.

## Footnotes

^1^http://ctdbase.org/

^2^https://jvenn.toulouse.inrae.fr/app/index.html

^3^http://www.genecards.org

^4^https://www.proteinatlas.org/

^5^https://mirtarbase.cuhk.edu.cn/~miRTarBase/miRTarBase_2022/php/index.php

^6^https://www.networkanalyst.ca/

^7^https://www.networkanalyst.ca/NetworkAnalyst/home.xhtml

^9^https://www.proteinatlas.org/humanproteome/immune+cell
